# Artificial Intelligence in Cardiovascular Imaging and Interventional Cardiology: Emerging Trends and Clinical Implications

**DOI:** 10.1016/j.jscai.2024.102558

**Published:** 2025-03-18

**Authors:** Maryam Alsharqi, Elazer R. Edelman

**Affiliations:** a Institute for Medical Engineering and Science, Massachusetts Institute of Technology, Cambridge, Massachusetts; b Cardiovascular Medicine, Brigham and Women’s Hospital, Harvard Medical School, Boston, Massachusetts

**Keywords:** artificial intelligence, cardiovascular imaging, deep learning, interventional cardiology, machine learning, transcatheter intervention

## Abstract

Artificial intelligence (AI) has revolutionized the field of cardiovascular imaging, serving as a unifying force that brings together multiple modalities under a single platform. The utility of noninvasive imaging ranges from diagnostic assessment and guiding interventions to prognostic stratification. Multimodality imaging has demonstrated important potential, particularly in patients with heterogeneous diseases, such as heart failure and atrial fibrillation. Facilitating complex interventional procedures requires accurate image acquisition and interpretation along with precise decision-making. The unique nature of interventional cardiology procedures benefiting from different imaging modalities presents an ideal target for the development of AI-assisted decision-making tools to improve workflow in the catheterization laboratory and personalize the need for transcatheter interventions. This review explores the advancements of AI in noninvasive cardiovascular imaging and interventional cardiology, addressing the clinical use and challenges of current imaging modalities, emerging trends, and promising applications as well as considerations for safe implementation of AI tools in clinical practice. Current practice has moved well beyond the question of whether we should or should not use AI in clinical health care settings. AI, in all its forms, has become deeply embedded in clinical workflows, particularly in cardiovascular imaging and interventional cardiology. It can, in the future, not only add precision and quantification but also serve as a means by which to fuse and link multimodalities together. It is only by understanding how AI techniques work, that the field can be harnessed for the greater good and avoid uninformed bias or misleading diagnoses.

## Introduction

Artificial intelligence (AI), machine learning (ML), and deep learning (DL) applications have revolutionized the field of cardiovascular medicine and have been used over a spectrum of applications from defining disease and intervention success to determining optimal patient selection and now driving interventions in semiautomated and fully automated fashions as well as in discovering hidden patterns and insights for personalized treatment. We review the noninterventional and interventional uses of AI separately below, fully cognizant that the future will meld all applications into a full-service whole in the near future.

What started decades ago principally in aiding angiography and transcatheter angioplasty^1^ has now absorbed all cardiovascular diseases and although percutaneous coronary intervention (PCI) remains the most common procedure, a range of other conditions are being percutaneously managed, including cardiac arrhythmias, valvular heart disease, congenital heart disease, pericardial disease, myocardial disease, and heart failure. With the expansion of transcatheter interventions, noninvasive cardiovascular imaging plays a critical role in guiding and facilitating such procedures. Noninvasive imaging modalities include cardiac ultrasound imaging (echocardiography), cardiac magnetic resonance imaging (MRI), nuclear imaging, and cardiac computed tomography (CT). Preoperative imaging allows accurate estimation of structural, morphological, and functional assessment, whereas peri-operative imaging offers cardiologists real-time guidance of intracardiac device positions and dynamic responses. These imaging modalities are also applied to evaluate outcomes and impact after interventional procedures.

The utility of noninvasive imaging ranges from diagnostic assessment, and guiding interventions, to prognostic stratification. Multimodality imaging has shown important potential specifically in patients with heterogeneous diseases, such as heart failure and atrial fibrillation. Facilitating complex interventional procedures requires accurate image acquisition and interpretation as well as precise decision-making. However, the traditional practice of image acquisition, analysis, and decision-making presents issues in timing, scaling, and diagnostic accuracy. AI and ML applications in cardiovascular imaging are rapidly gaining prominence as generated images become increasingly complex. AI algorithms have the capacity to streamline the clinical workflow through rapid and accurate image interpretation and quality control. They can help expand our understanding of certain interventions to improve clinical decision-making, particularly with the recent advances in stent technology and transcatheter valve replacement. This review focuses on the advancements of AI in noninvasive cardiovascular imaging related to interventional cardiology, tackling the clinical use and challenges of available imaging modalities, emerging trends, and promising AI applications as well as considerations for safe implementation of AI tools in clinical care settings. Recent applications across different interventions and noninvasive imaging modalities are described in Table 1.^2^, ^3^, ^4^, ^5^, ^6^, ^7^, ^8^, ^9^, ^10^

**Table 1:** Recent applications of AI techniques in noninvasive cardiovascular imaging and interventional cardiology.

Cardiac intervention and imaging modality	Reference, year	AI technique	Description	Sensitivity	Specificity	Accuracy	AUC
Coronary artery intervention							
Echocardiography	Upton et al,^2^ 2022	Automated image processing	To automate the detection of severe coronary artery disease from stress echocardiography images	0.844	0.927	–	0.93
Nuclear imaging	Mohebi et al,^3^ 2023	Prediction model	To predict postrevascularization ejection fraction	0.75	0.87	0.84	0.83
Cardiac CT	Griffin et al,^4^ 2023	Image segmentation and classification	To automate the detection and quantification of coronary artery stenosis from CCTA	0.94^a^ 0.94^b^	0.68^a^ 0.82^b^	0.84^a^ 0.86^b^	0.88^a^ 0.92^b^
Structural transcatheter intervention							
Echocardiography	Sengupta et al,^5^ 2021^c^	Prediction model	To augment the grading of aortic stenosis and optimize the timing of AVR	0.955	–	0.943	0.988
Cardiac MRI	Evertz et al,^6^ 2022	Automated quantification	To automate biventricular quantification and predict risk in patients undergoing TAVR	–	–	–	0.686
Cardiac CT	Chang et al,^7^ 2021	Automated image processing and quantification	To automate quantification of aortic valve calcium from cardiac CT scans	0.99	0.842	0.985	0.964
Electrophysiology ablation							
Echocardiography	Yuan et al,^8^ 2024	Image (video) processing and prediction	To identify patients with occult atrial fibrillation from TTE	0.69	0.68	0.68	0.96
Cardiac MRI	Shade et al,^9^ 2020	Prediction model	To estimate the risk of atrial fibrillation recurrence after pulmonary vein isolation	0.82	0.89	–	0.82
Mechanical support							
Echocardiography	Kanwar et al,^10^ 2018	Risk prediction	To predict mortality from preoperative clinical and imaging variables	0.28-0.33	0.87-0.9	0.76-0.87	0.7-0.71

AUC, area under the curve; AVR, aortic valve replacement; CCTA, coronary CT angiography; CT, computed tomography; MRI, magnetic resonance imaging; TAVR, transcatheter aortic valve replacement; TTE, transthoracic echocardiography.

a For the detection of ≥50% stenosis level.

b For the detection of ≥70% stenosis level.

c Involves multimodality imaging approach.

## AI techniques

AI encompasses a wide range of various techniques that can be applied in interventional cardiology for the development of diverse applications. The choice of the technique depends on the purpose of the model development. There has been a surge of interest, and an increased number of Food and Drug Administration–cleared applications in cardiovascular imaging spanning image processing, quality improvement, automated segmentations, and decision support tools. Table 2 summarizes the most commonly applied AI techniques in noninvasive cardiovascular imaging and interventional cardiology.

**Table 2:** AI techniques in noninvasive cardiovascular imaging and interventional cardiology.

AI technique	Applications	Examples
Image processing and generation	Enhances image quality, generates additional images, and addresses imaging challenges (eg, lengthy acquisition time, artifacts, radiation exposure).	- Undersampling for faster cardiac MRI scans. - Virtual image creation to lower radiation dose (eg, contrast-enhanced CT from nonenhanced images). - GAN for cine-like cardiac MRI synthesis.
Image classification and segmentation	Automates image analysis to improve accuracy and reproducibility, saving physicians time and effort. Includes steps like classification of views and segmentation of cardiac anatomy.	- Multiscale deep reinforcement learning for anatomical landmark detection. - U-Net and CNN-based methods for segmentation of cardiac chambers, valves, and vessels. - Quality control frameworks to reduce interpretation time.
Association and prediction	Identifies patient-specific clinical associations and predicts outcomes by analyzing complex data sets, integrating clinical symptoms, imaging data, and biomarkers.	- SVM and random forests for precision medicine. - Bayesian networks for early cardiovascular event prediction. - XGBoost and random forests outperforming logistic regression for survival predictions.
Decision support system	Provides patient-specific summaries and risk assessments using electronic health records and imaging data. Incorporates clinical decision support for cardiac interventions.	- NLP for personalized decision support. - Integration of imaging findings and clinical data for tailored recommendations.

CNN, convolutional neural network; CT, computed tomography; GAN, generative adversarial networks; MRI, magnetic resonance imaging; NLP, natural language processing; SVM, support vector machines.

### Image processing and generation

The scope of AI applications in image processing focuses mainly on enhancing image quality and generating additional images to overcome traditional imaging challenges such as lengthy acquisition time, imaging artifact, and radiation exposure while maintaining interpretable image quality.^11^ Undersampling is one technique that has significantly accelerated cardiac MRI scan times by acquiring fewer images and estimating the remainder.^12^ Computer vision for virtual image creation is another approach to lower radiation dose by reducing the number of acquired imaging series. It allows contrast-enhanced CT images to be created from nonenhanced images and vice versa.^13^ Generative adversarial networks have the capacity to synthesize cine-like cardiac MRI from real-time sequences, helping patients with arrhythmia or those who struggle during breath-holding image acquisition, resulting in improved image quality.^14^

### Image classification and segmentation

Automated image analysis is 1 of the primary goals of using AI in imaging as it can save imaging physicians time and effort as well as increase the accuracy and reproducibility of image analysis. Automated analysis involves multiple preprocessing steps to achieve optimal performance including image classification and segmentation algorithms. Identification of standard cardiac views is an essential step that is performed by detecting specific features and anatomical landmarks that are unique to a specific view angle (ie, differentiating a long-axis view from a short-axis view of the heart). Disease classification has been also of interest to differentiate cardiac images of physiologic from pathologic left ventricular patterns.^15^ Researchers have recently shown that using a multiscale deep reinforcement learning approach allowed accurate anatomical landmark identification of 5000 3D cardiac CT images (2,500,000 slices) in less than a second.^16^^,^^17^

After image classification, the following step is anatomical segmentation which is crucial in the quantification of cardiac chambers, valves, and vessels. Automated segmentation models are often based on DL methods, such as U-Net architecture, that allows object detection, motion tracking, and chamber cavity or vascular wall contouring. Recent applications include a framework of multiple convolutional neural networks (CNN) for automated segmentation of anatomical structures and detection of segmentation failure areas in cardiac MRI.^18^ This framework revealed high performance with quality control of cardiac segmentations that have reduced the interpretation time by 10-fold.

### Association and prediction

As the computing power of AI has the capacity to handle big and complex data sets, discovering statistical associations, underlying physiological interactions, and meaningful clinical insights has become more feasible. Association analysis methods, such as support vector machines and random forests, facilitate the promise of precision cardiovascular medicine through the identification of patient-specific clinically relevant associations by integrating clinical symptoms, imaging data, and various biomarkers.^19^^,^^20^ Using a Bayesian network, findings from stress echocardiography and coronary angiography were integrated to understand underlying relationships and early predict adverse cardiovascular events.^21^ Regression-based prediction models and clinical prognosis applications can be developed using hybrid DL methods or ensemble boosting such as XGBoost. By training ML prediction models on 331,317 echocardiography scans, Samad et al^22^ have presented that nonlinear random forest models outperform logistic regression (*P* < .01) in the prediction of survival durations.

### Decision support system

The use of AI-assisted clinical decision systems has been of interest in the last few years. These systems use information from electronic health records and map imaging findings, clinical examinations, and admission visits. They present an outline of patient-specific summaries and notifications of risk factors, possible drug interactions, or relevant information for specific procedures such as cardiac catheterization. With the advancement of AI techniques, particularly in natural language processing, clinical decision support systems have become more flexible and can be customized to fit the needs of patients and clinical care teams.^23^

The key highlight from both Tables 1 and 2 is the extensive role of AI in cardiovascular imaging and its incredible impact on cardiovascular interventions. Within this framework, it is essential to consider each method in relation to its primary indication. As each method offers unique insight and should be evaluated individually, direct side-by-side comparison is therefore limited.

## The role of AI in noninvasive cardiovascular imaging related to interventional cardiology

Noninvasive cardiovascular imaging is essential in guiding transcatheter interventions, whether using echocardiography and MRI for structural and functional assessment, nuclear imaging for perfusion, or CT for anatomical visualization. Although each modality has a proven unique diagnostic and prognostic value, multimodality imaging approaches have been recommended particularly in patients with heterogeneous diseases, offering accurate planning and execution of interventional procedures and prognostic stratifications of clinical outcomes.^24^ However, the growing volume of images per scan complicates analysis, increasing errors and challenging human interpretation. AI, particularly DL techniques, can address such challenges by performing a high level of image processing and interpretation reducing human error, missing diagnosis, and variability. Examples of potential AI opportunities are listed for each imaging modality in the Central Illustration.

**Central Illustration:**
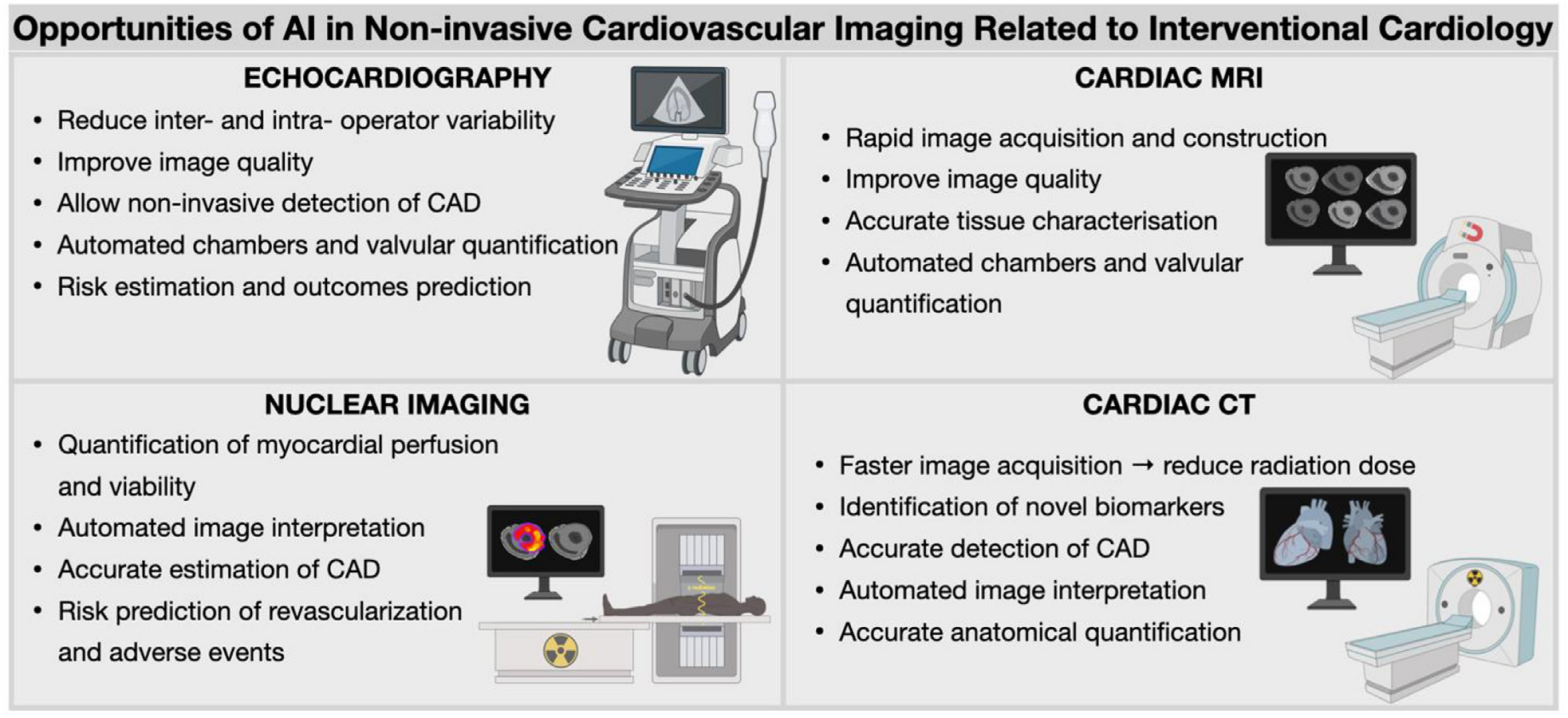
**Potential role of artificial intelligence (AI) in noninvasive cardiac imaging related to interventional cardiology.** CAD, coronary artery disease; CT, computed tomography; MRI, magnetic resonance imaging.

### AI in echocardiography

Echocardiography is the first-line noninvasive imaging modality for the assessment of cardiovascular structure and function and plays a key role in guiding interventional cardiology and therapeutic decisions. It is widely available, radiation-free, and offers bench-side real-time imaging of the heart with high temporal resolution. However, the image quality and interpretation are highly dependent on the operator’s skills, patient position, and anatomical visibility of the heart. Although applications of AI in echocardiography were introduced in the 1970s, there has only been recently a surge of interest in the development and implementation of clinical applications.

Machine learning and DL techniques now create a comprehensive pipeline for echocardiography automated analysis that encompasses view classification, anatomical segmentation, structural and functional quantification, and detection of pathological features.^25^ These techniques have already been implemented in several echocardiography laboratories and have been crucial in improving daily practice. Image quality enhancement (harmonic imaging) is an example of the use of AI algorithms for noise reduction and quality enhancement that are built-in in echocardiography machines. Automated quantification algorithms have also been installed on recent machines for automated calculations of fundamental structural and functional measures, such as ventricular volumes, ejection fraction, and longitudinal strain, offering mechanistic insights into myocardial diseases while maintaining a high level of reproducibility.^26^ Several applications focused on image classification and automated quantification in combination with clinical data or other imaging modalities to personalize the decision-making process.^25^ In a blinded and randomized trial, an AI-guided workflow of left ventricular ejection fraction assessment showed faster and more consistent analysis in comparison to manual assessment by sonographers.^26^ The feasibility of automated quantification and analysis of coronary artery disease (CAD) from stress echocardiography^2^ and detection of heart failure with preserved ejection fraction^27^ have been developed using AI with high accuracy, sensitivity, and specificity. A recent application of ML cluster analysis, using echocardiography images post transcatheter aortic valve replacement (TAVR) revealed 3 unique phenotype patterns highly associated with prognostic outcomes.^28^ Similarly, ML models were developed for the prediction of postoperative adverse events from baseline (preoperative) echocardiography images in patients with atrial fibrillation,^29^ and patients with transcatheter aortic valve replacement.^30^

### AI in cardiac MRI

Cardiac MRI is considered the gold standard imaging modality for noninvasive quantification of ventricular volumes and ejection fraction. Tissue characterization, perfusion, and viability of the myocardium can also be assessed using the late gadolinium enhancement (LGE) techniques offering high spatial resolution. Cardiac MRI is often performed before interventional procedures to precisely plan appropriate management, whereas during procedure imaging allows guidance for complicated interventions, particularly chosen to minimize the risk of radiation. Although cardiac MRI provides high-quality images, ventricular function quantification can be time and labor-intensive.

AI techniques are instrumental in addressing the lengthy cardiac MRI acquisition and processing time. Such techniques support image acquisition and preprocessing in the form of enhancement of image quality, elimination of artifacts, and automated sequence optimization to reduce scanning time. Postprocessing techniques involve automated classification, segmentation, and quantification for pathology detection that offer a time-effective and consistent analysis approach. DL techniques, such as undersampling reconstruction, have accelerated the image acquisition time by 2- to 3-fold by acquiring a small set of images and constructing the nonacquired images from existing data.^12^ Generative networks have been used to cine-MRI from real-time sequences improving image quality acquisition, particularly in patients with difficulty in breath-holding or arrhythmias.^14^ Furthermore, Ruijsink et al^31^ have demonstrated the role of CNN in processing large data sets of cine cardiac MRI without clinician interaction for automated structural and functional quantification.

### AI in nuclear imaging

Myocardial perfusion imaging is vital in nuclear medicine and offers crucial diagnostic and prognostic insights in CAD. Myocardial viability and perfusion defects can be assessed using single photon emission computed tomography (SPECT). Applications of ML algorithms with SPECT have been particularly focused on integrating clinical information along with imaging for automated detection, prediction, and risk stratification of CAD. Such applications can be specifically useful in guiding interventionists for personalized clinical management as well as in the patient selection process for high-risk coronary interventions.

Automated interpretation of myocardial perfusion imaging using a DL algorithm has shown improvement in the sensitivity of predicting obstructive CAD compared to the total perfusion deficit.^32^^,^^33^ The diagnostic accuracy and detection of myocardial perfusion defects in SPECT were also improved in >1000 patients with CAD when ML techniques were applied.^34^ Using a boosted ensemble ML algorithm to integrate SPECT images and clinical data demonstrated higher accuracy in predicting CAD compared to total perfusion deficit and visual analysis by 2 experts. Similarly, Arsanjani et al^35^ have reported accurate predictions of early revascularization in patients with suspected CAD using a supervised ML algorithm to combine quantitative features from SPECT imaging and clinical data. Moreover, the combination of clinical data with SPECT imaging using ML techniques offered an important prognostic value in risk estimation of cardiac death,^36^ and prediction of major adverse cardiovascular events in a 3-year follow-up duration.^37^

### AI in cardiac CT

Cardiac CT, primarily used with coronary computed tomography angiography (CCTA), enables noninvasive 3D visualization of coronary vasculature and detection of coronary artery calcification. It is the imaging modality of choice for rapid and accurate assessment of cardiovascular vasculature specifically for patients with suspected CAD and aortic dissection. AI applications in cardiac CT aim to minimize radiation exposure and reduce image acquisition time while producing interpretably high image quality.

Similar to MRI, AI techniques have demonstrated substantial advancements in both the diagnostic and prognostic capabilities of CCTA.^38^ Recently, a fully automated DL pipeline has been developed based on a range of U-Net architectures for comprehensive assessment and characterization of coronary arteries.^39^ This pipeline demonstrated high accuracy in automated coronary artery segmentation and quantitative assessment of coronary artery calcium and tortuosity that could be used for personalized risk assessment prior to PCI. In addition, the advancement of functional assessment of coronary lesions through calculating the fractional flow reserve (FFR) has been of interest. Using a multiscale CNN algorithm, Zreik et al^40^ have developed an automated tool for left ventricular myocardial segmentation for noninvasive estimation of FFR which demonstrated good agreement with invasive FFR measurements. Furthermore, results from a multicenter registry of patients who underwent CCTA with a minimum of 3-year follow-up duration demonstrated that ML-derived risk scores provided greater prognostic accuracy compared to traditional CCTA risk scores.^41^ More recently, an ML application in the quantification of coronary artery calcium score has demonstrated promising results that were well-aligned with expert interpretation.^42^

## Opportunities of AI and cardiovascular imaging in interventional cardiology

Although applications of AI in cardiovascular imaging have led to transformational shifts, their role in interventional cardiology is still in an early stage. Multimodality imaging is important in facilitating major cardiovascular interventions for ischemic, structural, electrophysiological, and mechanical cardiovascular diseases. Successful cardiovascular interventions require accurate and precise noninvasive assessment prior to the intervention for management planning, during the intervention for catheter and device guiding, and post the intervention to assess outcomes and complications.

### Coronary artery interventions

PCI is the most commonly performed intervention and is used in the first-line management of patients with myocardial infarction and ischemic heart diseases. Although opportunities for AI in PCI have not been fully explored, recent studies have demonstrated potential applications in the near future. Preinterventional noninvasive imaging can also help in predicting the risk of target vessel revascularization or subsequent stent failure, especially when integrated with AI. Although the advent of virtual FFR systems in AI-guided angiography has allowed 3D reconstruction of coronary arteries to quantify stenotic lesions, it still requires an invasive intervention.^43^ Recent advancements in CCTA and AI algorithms have enabled noninvasive estimation of FFR by 3D reconstruction of coronary arteries to identify hemodynamically significant lesions.^4^^,^^44^ In a comparison of the detection and grading of coronary artery stenosis between invasive FFR, coronary angiography FFR, and AI-derived FFR from CCTA, AI-derived FFR showed high sensitivity and accuracy for severe degree of stenosis at both ≥50% and ≥70%. The AI-derived FFR analysis was performed with an average time of 10.3 ± 2.7 minutes, allowing rapid and accurate noninvasive assessment.^4^ Thus, the emergence of preinterventional CCTA could positively shift the current practice, for PCI in particular, by enabling more accurate diagnoses, guiding vascular access selection, optimizing fluoroscopy viewing angles, and planning procedural strategies.^45^ With such approaches, interventionists will be equipped with a detailed procedural roadmap, reducing complications and adverse events.

Additionally, an ML prediction model for postrevascularization ejection fraction from pre-PCI myocardial perfusion SPECT imaging has been recently proposed.^3^ Seven classifiers were implemented on 1700 features extracted from each SPECT scan to predict the change in ejection fraction response after revascularization. Using echocardiography, ejection fraction was measured pre- and post-PCI, and its change was predicted in 3 classes: reduction or no improvement (class 1), up to 5% improvement (class 2), or more than 5% improvement (class 3). In addition, from a cohort of 9680 patients who underwent PCI, 6 ML models were developed to integrate pre-PCI clinical information and echocardiography findings for clinical outcomes prediction.^46^ On a larger-scale study, using clinical information and ejection fraction from >35,000 patients across 4 continents, ML risk assessment models were developed to predict target lesion failure post-PCI.^47^ Such ML models can help to assist clinicians in the patient selection process and identify those who will benefit from the PCI.

### Structural heart transcatheter interventions

The current practice of preprocedural planning for precise selection of the size and type of transcatheter devices to meet patients’ needs is time- and expertise-demanding.^48^ A recent study has tested the use of fully automated functional assessment in comparison with manual assessment of cardiac MRI for risk stratification prior to TAVR intervention.^6^ Both approaches showed comparable prediction accuracy of cardiovascular mortality, but the automated approach saved at least 10 minutes per patient of the assessment time. Similar findings have been reported to support that automated quantification of valvular morphology using AI provided faster and more accurate measurements compared to manual quantification offering efficient perioperative planning.^49^^,^^50^ Using ML techniques, mitral valve repair biomechanics were simulated to compute patient-specific mitral valve apparatus from transesophageal echocardiography (TEE) images. Promising results were found when comparing the predicted mitral valve closure in the simulation model with real outcomes.^51^ Several studies have demonstrated that the use of image-based simulation models of patient-specific anatomy offered efficient preprocedural planning, in terms of device type and size selection as well as depth of implantation.^52^, ^53^, ^54^, ^55^

Machine learning methods can enhance the implementation of simulation models in clinical practice as guidance tools for complicated or rarely performed procedures as well as guiding junior interventionists.^56^ For more realistic visualization of real-time anatomy during transcatheter valve interventions, HoloLens (Microsoft) offers a mixed-reality display of 3D TEE images of the mitral valve visible as a semitransparent holographic cube conveniently positioned for interventionists.^57^ A similar approach has also been developed using 3D CTA images to display holograms in mixed reality during percutaneous patent ductus arteriosus closure.^58^ Furthermore, Miller et al^59^ proposed a feasible robotic-assisted MRI to guide TAVR procedures, offering real-time visualization of device deployment.

Perioperative fusion of noninvasive imaging and fluoroscopy techniques allows automated identification of anatomical landmarks for correct valve or device implantation. TEE and fluoroscopy fusion resulted in decreased radiation exposure time when used to guide left atrial appendage closure^60^ and TAVR interventions.^61^ More recently, Bavo et al^62^ validated the performance of a CT-based computational model to optimize percutaneous left atrial appendage closure interventions. Using patient-specific anatomical models of the appendage and simulating device apposition, the optimal device size and positioning deformations were identified allowing precise noninvasive preinterventional planning.

Using supervised ML models, postintervention complications and in-hospital mortality have been predicted at an area under the curve (AUC) of 0.92 for patients undergoing TAVR,^63^ and 0.83 for mitral valve repair.^64^ The advancement and implementation of such models will transform the current practice of transcatheter interventions by minimizing device-related complications and offering early prediction of adverse outcomes.

### Electrophysiological cardiac interventions

AI applications have been adopted in the field of electrophysiology for decades, including signal analysis, arrhythmia detection, automated signal interpretation, and pacemaker algorithms.^65^ However, the recent growth of DL techniques and computational power have unlocked a new path of expansion in multimodal integrative approaches for the noninvasive characterization of arrhythmias. Myocardial tissue characterization using cardiac MRI and ML techniques aids high-precision noninvasive localization of arrhythmia foci.^66^ Moreover, scarred myocardial regions identified in LGE images form the basis of ablation strategies such as scar dechanneling and homogenization in ventricular tachycardia ablation.^65^ Fahmy et al^67^, ^68^, ^69^ have developed DL-based automated approaches to quantify myocardial scar tissue using CNN. These approaches have also aided pulmonary vein isolation procedures in atrial fibrillation.^65^ Using a multimodal integrative approach with ML techniques, Shade et al^9^ have conducted a proof of concept study to develop a preprocedural personalized prediction model for the assessment of the probability of atrial fibrillation recurrence post pulmonary vein isolation. Prediction features were derived from tissue characteristics from LGE MRI, and atrial fibrillation simulations through a computational model. Using random forests for unbiased feature selection, the model performance achieved an AUC of 0.82 in predicting atrial fibrillation recurrence. The results of these studies are promising paving the way for new concepts in the field of electrophysiology but require further validation in larger clinical trials.

The recent advancements in noncontact magnetic and ultrasound navigated robotic ablation systems have enhanced the success rate of catheter ablations as well as significantly reduced patient exposure time to fluoroscopy.^70^, ^71^, ^72^ In a randomized study of 80 patients with symptomatic atrial fibrillation, pulmonary vein isolation procedures with a robotic navigation system offered higher contact force, lower rate of recurrence, and reduced fluoroscopy time.^73^ ML-aided catheter ablation procedures have also demonstrated the feasible potential of improved real-time guidance and voltage mapping through an active-learning model.^74^ Although other AI applications in electrophysiology treatment planning and procedural assistance have shown transformative potential in the field,^75^ this review focuses solely on imaging-related applications.

### Mechanical support

The implantation of percutaneous transcatheter mechanical circulatory support (MCS) devices has increased rapidly in patients with severe heart failure, cardiogenic shock, or high-risk PCI. Outcome variability and appropriate patient selection have been major limitations of these devices. AI applications of integrating clinical data with imaging have shown promising potential in identifying patient suitability for specific devices, evaluating the need for additional support, monitoring complications, and predicting mortality rates. Quantification of ventricular function prior to MCS device implantation is often performed using echocardiography or cardiac MRI for higher accuracy, specifically for the right ventricle. Fully automated analysis using ML algorithm to quantify right ventricular volumes and function from echocardiography images has allowed the development of comparable diagnostic performance to cardiac MRI with higher reproducibility compared to manual analysis.^76^ Right ventricular dysfunction is a critical complication of left ventricular MCS.^77^ The incidence of acute, early, and late right ventricular failure post-MCS device implantation was predicted with AUC between 0.83 to 0.90, using Bayesian models on a large multicenter registry (Interagency Registry for Mechanically Assisted Circulatory Support).^78^ Using the same data set, a study demonstrated that using a Bayesian model has enabled prediction of mortality rate following left ventricular assist device.^10^ Preimplantation left ventricular end-diastolic diameter measured from echocardiography images was 1 of the predictor parameters of 1-, 3-, and 12-month mortality. Because these approaches are relatively recent, their clinical implications have not been fully studied.

Increasingly, AI will be used to drive and operate MCS—everything from ventilators to renal replacement therapy to cardiovascular support. Indeed, the controllers of percutaneous left ventricular devices incorporate AI and ML to extrapolate hemodynamic effects and, in the future, determine weaning protocols and advancing therapies.^79^^,^^80^

## Challenges and future implications

Although there is a growing body of literature on the use of AI and ML models in cardiovascular imaging related to interventional cardiology claiming superiority to traditional methods, few of these applications have yet progressed and been scaled to impact clinical outcomes.^81^ Wilkinson et al^82^ have demonstrated that only 24% of published AI algorithms have been tested on external cohorts, whereas the remaining majority do not validate the algorithm performance in different settings. For instance, multiple ML-based models have been developed to predict the rehospitalization rates or adverse outcomes in heart failure, but only limited improvement was achieved.^83^^,^^84^

**Figure 1:**
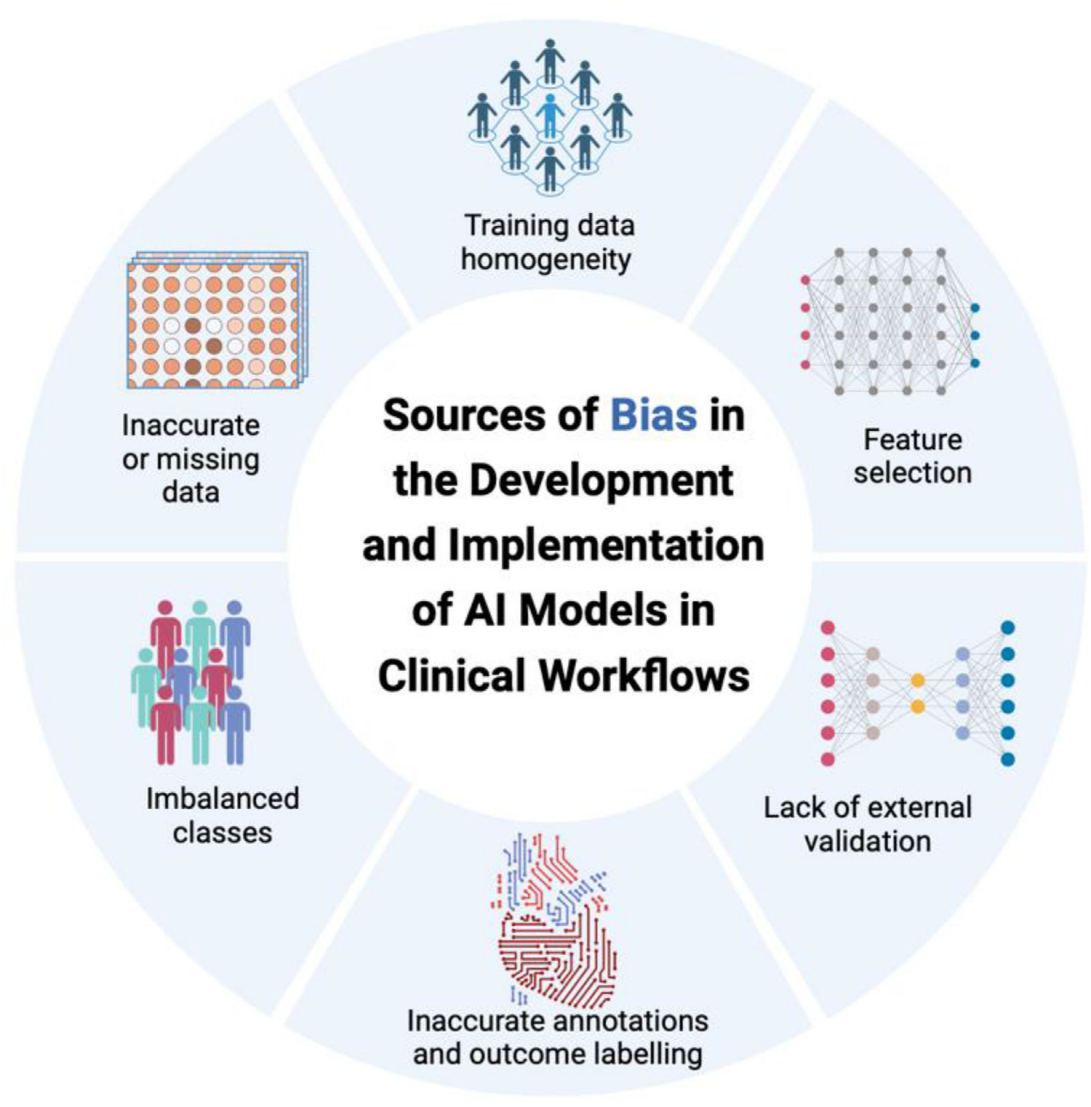
**Possible sources, consequences, and mitigation strategies of bias in the development and implementation of artificial intelligence (AI) models in clinical workflows**.

**Figure 2:**
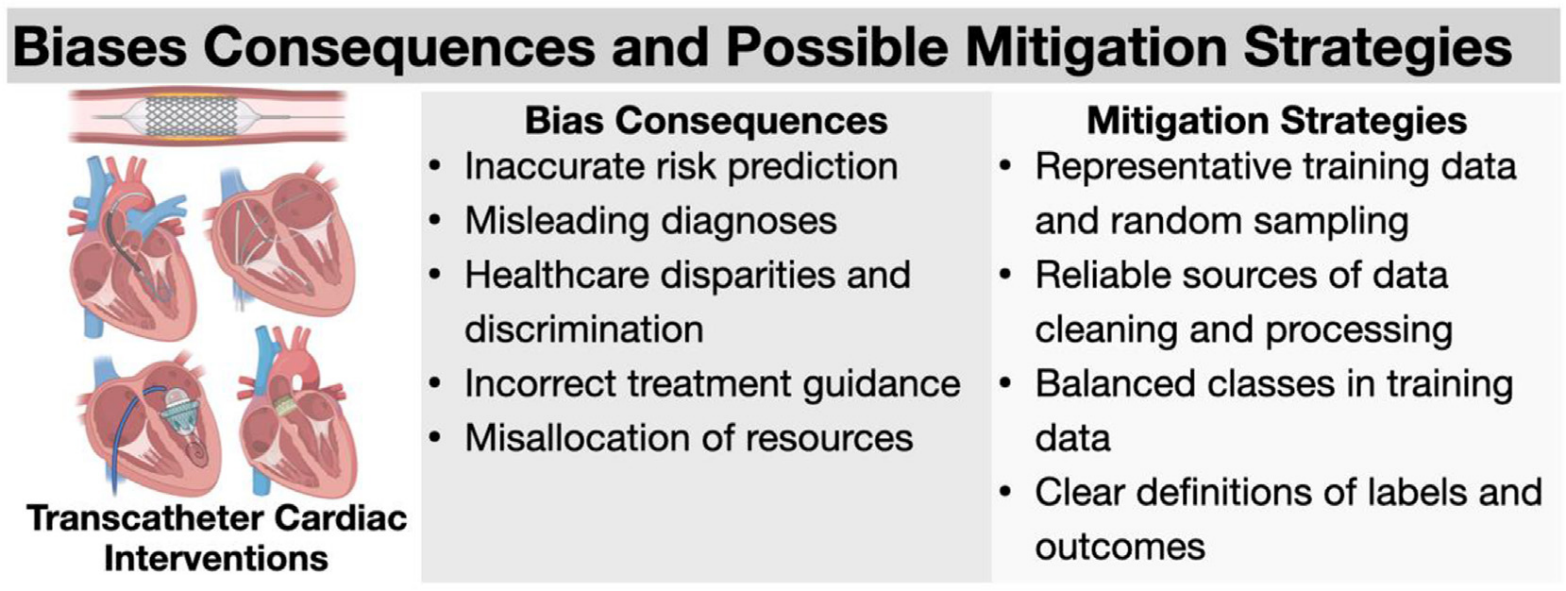
**Bias consequences and possible mitigation strategies in the development and implementation of artificial intelligence models in cardiovascular interventions**.

Major challenges of implementing AI applications in clinical workflows are the inherited bias from the model development process and ethical concerns related to autonomous decisions for patients’ health care. Biases can be introduced at multiple levels of the pipeline of AI model development, validation, and implementation.^85^ Training a model on imbalanced data sets, leads to unfair or false decisions about underrepresented populations.^86^^,^^87^ Underrepresentation of women and minorities (sex, ethnic, racial, and economic biases) in training data sets will cause inaccurate generalization of model performance.^88^ The lack of standard performance metrics, inconsistent annotations, and the size of training data sets can also introduce unintentional biases into the model development process. Possible sources of bias in the development and implementation of AI models in clinical workflows are illustrated in Figure 1. Figure 2 illustrates examples of bias consequences and potential mitigation strategies.

Ethical concerns related to clinical implementation of AI models, include challenges in maintaining patient confidentiality and lack of authorities to govern AI standardization. As the clinical implementation process requires the exchange of patient clinical and imaging data across multiple institutions and countries, it may result in breaches of patient privacy and confidentiality.^89^ In addition, AI-related errors could raise ethical and legal considerations for accountability, particularly with fully autonomous AI models, in which the legal liability becomes more complex. Currently, even with fully automated image quantification models, AI-generated outcomes are reviewed and confirmed by clinicians before making clinical decisions.^19^ However, in the future, with the rapid growth of implementing AI models in cardiovascular imaging, some steps of the analysis are likely to be autonomous and may evolve to complete human operator independence by necessity of programmed and intended operation. In such instance, who becomes responsible for incorrect quantification causing unintended fatal complications during AI-guided cardiovascular intervention, the interventionist, the imaging specialist, the responding clinician, or the model developer? Moreover, with an AI-guided decision-making model resulting in bias amplification, who will be responsible to mitigate or lead these decisions?^90^ To minimize the effect of biases in AI model development and promote safe practice, the Food and Drug Administration, Health Canada, and the United Kingdom’s Medicines and Healthcare products Regulatory Agency have jointly identified 10 guiding principles for developing good ML applications.^91^ The principles are summarized in Table 3.^91^ AI- and ML-based applications can transform clinical workflows in noninvasive imaging and interventional cardiology, and offer promising applications for precision medicine, assisting with the diagnosis and treatment selection process, preprocedural planning, execution procedures, and outcomes prediction, only if used with awareness of potential bias and ethical considerations.

**Table 3:** Guiding principles for good machine learning practice for medical devices.^91^

Principle	Description
1. Multidisciplinary expertise	Deep understanding of the integration process of a model into clinical workflow, including both the clinical benefits and associated risks.
2. Software engineering and security practice	Clinical implementation of a model design following good practices of software engineering, cybersecurity, data management, and quality assurance.
3. Representation of the intended patient population	Sufficient representation of clinical study participants with adequate sample size for training and testing data sets to allow outcomes generalizability to the population of interest.
4. Independent test set from the training set	Selection of independent data sets for training and testing considering all sources of dependence.
5. Best available methods for reference data set	Reference data sets are developed based on accepted and best available methods.
6. Model design suitability for the available data and intended use	Model design is tailored to the available data to achieve the desired benefits safely and effectively.
7. Evaluation of the human-AI team performance	Addressing human involvement in the model outputs with a focus on human-AI team performance
8. Testing a model performance during clinically relevant conditions	Statistical test plans for a model performance on clinically relevant conditions and independent of the training data set.
9. Provide clear essential information to users	Users are provided with clear ready access to essential information appropriate for the intended audience, including indications for use, performance and clinical benefits for intended populations and specific subgroups, known limitations, and potential associated risks.
10. Performance monitoring and risk management	Monitoring of deployed models in real-world performance with maintained and improved safety measures in place to manage risks of overfitting, unintended bias, and degradation of the model.

## Conclusion

Current clinical practice is well beyond the point of whether we should or should not use AI applications in clinical health care settings. AI in all its forms has been deeply embedded in clinical workflows, particularly in cardiovascular imaging and interventional cardiology. AI, in fact, serves as a unifying force especially in imaging, in which it brings together disparate modalities under a single platform. It can, in the future, not only be used to add precision and quantification but also as a means by which to fuse and link multimodalities together. It is only by understanding how AI techniques work, that the field can harness it for the greater good and avoid uninformed bias or misleading diagnoses.
